# STING activation normalizes the intraperitoneal vascular-immune microenvironment and suppresses peritoneal carcinomatosis of colon cancer

**DOI:** 10.1136/jitc-2020-002195

**Published:** 2021-06-18

**Authors:** Seung Joon Lee, Hannah Yang, Woo Ram Kim, Yu Seong Lee, Won Suk Lee, So Jung Kong, Hye Jin Lee, Jeong Hun Kim, Jaekyung Cheon, Beodeul Kang, Hong Jae Chon, Chan Kim

**Affiliations:** 1 Department of Biomedical Science, CHA University, Seongnam, Gyeonggi-do, Korea (the Republic of); 2 Medical Oncology, CHA Bundang Medical Center, CHA University School of Medicine, Seongnam, Gyeonggi-do, Korea (the Republic of); 3 Department of Surgery, CHA Bundang Medical Center, Seongnam, Gyeonggi-do, Korea (the Republic of)

**Keywords:** immune evation, immunotherapy, interferon inducers, tumor microenvironment, immune reconstitution

## Abstract

**Background:**

Peritoneal carcinomatosis is a fatal clinical presentation of colon cancer, characterized by unresponsiveness to conventional anticancer therapies, including immune checkpoint inhibitors. Here, we elucidated the immune-evasion mechanisms during the peritoneal carcinomatosis of colon cancer and developed a novel immunotherapy by activating the stimulator of interferon genes (STING) pathway.

**Methods:**

We generated a syngeneic peritoneal carcinomatosis model of colon cancer. Mice were intraperitoneally treated with either STING agonist (MIW815, also known as ADU-S100) or PD-1 blockade or both. The tumor microenvironment was comprehensively analyzed using multiplexed immunofluorescence imaging, flow cytometry, and NanoString immune profiling.

**Results:**

Intraperitoneal colon cancer cells induce a massive influx of immunosuppressive M2-like macrophages, upregulate immune checkpoints, and impair effector T cell functions during peritoneal dissemination; these collectively create a highly angiogenic and immunosuppressive milieu that is resistant to anti-PD-1 monotherapy. Intraperitoneal administration of a STING agonist suppressed aberrant angiogenesis, increased pericyte coverage, and normalized tumor vessels, thereby facilitating the infiltration of activated CD8^+^ T cells into peritoneal tumor nodules. Moreover, STING activation reprogramed tumor-associated macrophages toward the M1 phenotype. STING activation converted immunologically cold peritoneal tumors into T-cell-inflamed tumors in a type-I interferon-dependent manner. Lastly, the STING agonist synergistically cooperated with PD-1 and/or COX2 blockade to further suppress the peritoneal dissemination of colon cancer, resulting in complete eradication of tumor and ascites, and inducing durable antitumor immunity.

**Conclusions:**

STING activation can normalize the peritoneal vascular and immune microenvironment, providing a rationale for a novel combination therapeutic strategy for peritoneal carcinomatosis in colon cancer.

## Introduction

Peritoneal carcinomatosis of colon cancer (PCCC) is an uncontrolled dissemination of cancer cells into the peritoneal cavity that is usually considered an end-stage manifestation of the disease.^1 2^ PCCC is known to have poorer prognosis compared with other sites of metastasis.^2 3^ Local and systemic therapies, including immunotherapies, show limited efficacy, making PCCC a major therapeutic challenge that hampers the survival and quality of life of patients with colon cancer.^3–6^

The progression of PCCC is promoted by robust tumor angiogenesis, which generates tortuous and chaotic networks of neovessels.^7 8^ Because these immature vessels are loosely connected and lack adequate pericyte coverage, they are hyperpermeable. This allows the extravasation of massive amounts of plasma fluid and tumor cells into the peritoneal cavity, thus accumulating malignant ascites.^2 8^ Moreover, this vasculature cannot provide consistent blood flow into the peritoneal tumor nodules,^9^ thereby disturbing the efficient delivery of anti-cancer drugs and immune effector cells into the tumor microenvironment (TME).^2 10^ Another important driver of PCCC is tumor-induced immune suppression within peritoneal cavity.^4 11^ In physiological condition, the peritoneal cavity secures immunologic competence against diverse pathogens with abundant dendritic cells, T cells, and various soluble factors in the peritoneal fluid.^11 12^ However, during peritoneal progression of colon cancer, tumor cells evade immunological recognition and destruction, enabling their own survival and rapid dissemination within the peritoneal cavity.^13 14^ Although this peritoneal immune suppression is a decisive step during peritoneal dissemination of colon cancer, the underlying mechanism is poorly understood.

Stimulator of interferon genes (STING) is an innate immune sensor that detects the presence of cytosolic DNA and serves as an important link between innate and adaptive immunity within the tumor.^15–18^ The activation of the STING signaling cascade elicits robust type-I interferon (IFN) responses that activate dendritic cells and stimulate cross-priming of tumor neoantigens to CD8^+^ T cells.^19–23^ Moreover, STING signaling is also involved in the regulation of the tumor vasculature in various malignancies and can stimulate tumor endothelial cells to secrete type-I IFNs, triggering spontaneous and therapeutic antitumor immunity within the TME.^24–27^ Additionally, recent studies unveiled that intratumoral STING activation can remodel the phenotype of the tumor vasculature to enhance endothelial-lymphocyte interactions and facilitate intratumoral trafficking of CD8^+^ T cells.^24 27 28^

Here, we elucidated the establishment of a highly angiogenic and immunosuppressive milieu within the peritoneal cavity during peritoneal dissemination of colon cancer. This unfavorable TME was overcome by intraperitoneal STING activation, which suppressed tumor angiogenesis and rejuvenated peritoneal immunity. These subsequently led to an efficient reduction in the number of peritoneal metastases and malignant ascites volume.

### Material and methods

### Mice and cell line

C57BL/6 mice (7–8 weeks old) were purchased from Orient Bio (Seongnam, Korea). All mice were maintained in a specific pathogen-free animal facility at CHA University (Seongnam, Korea). MC38 colon cancer cell line and ID8 ovarian cancer cell line was obtained from National Cancer Center (Goyang, Korea). The cells were maintained at 37°C with 5% CO_2_ in Dulbecco’s modified Eagle’s medium (DMEM) (Biowest) supplemented with 10% fetal bovine serum and 1% penicillin/streptomycin.

### Tumor models and treatment regimens

MC38 colon cancer cells (5×10^5^) were intraperitoneally injected into the peritoneal cavity of male C57BL/6 mice to generate peritoneal tumors, as previously described.^8^ ID8 ovarian cancer cells (1.5×10^7^) were intraperiotneally injected into female C57BL/6 mice. The mice were then administered 15 µg of the STING agonist, dithio-(Rp, Rp)-2′,5′-3′,5′-c-diAMP sodium salt (RR-CDA, also known as MIW815 or ADU-S100, Invivogen) by intraperitoneal injection at indicated time points in the figures. The control group was treated with the same amount of phosphate-buffered saline (PBS) instead of the STING agonist. For the T cell depletion study, each depletion group received intraperitoneal injections of anti-IFN α receptor (IFNAR) (200 µg, clone MAR1-5A3, BioXCell), anti-CD8a (200 µg, clone 53–6.7, BioXCell), or anti-CD4 (200 µg, clone GK1.5, BioXCell) antibody at 2 or 3-day intervals. For combination therapy, we performed intraperitoneal injection of anti-programmed cell death protein 1 (PD-1) antibody (250 µg, clone J43, BioXCell) at a given time point in the figures. To inhibit the indoleamine 2,3-dioxygenase (IDO) or cyclooxygenase-2 (COX2) pathway, each inhibitory group was orally administered with epacadostat (100 mg/kg, LEAPChem) or celecoxib (60 mg/kg, Sigma) daily for 10 days.

### Immunofluorescence

For immunofluorescence staining, the tumor tissues were fixed in 1% paraformaldehyde at room temperature (25°C), dehydrated in 20% sucrose overnight, and embedded in tissue-freezing medium (Leica). The frozen samples were sectioned into 20-μm-thick slices, permeabilized with 0.3% Triton X-100 in PBS (PBST) for 3 min at room temperature, and processed with blocking solution (5% normal goat serum in 0.1% PBST) for 1 hour. The samples were incubated at 4°C overnight with the following primary antibodies: anti-CD8 (rat, clone 53–6.7, BD Bioscience), anti-CD4 (rat, clone RM4-5, Invitrogen), anti-CD31 (rabbit polyclonal, Abcam), anti-caspase3 (rabbit polyclonal, R&D System), anti-CD11b (rat, clone M1/70, BD Biosciences), anti-F4/80 (rabbit polyclonal, Abcam), anti-CD206 (rat, clone MR5D3, Invitrogen), anti-iNOS (rabbit polyclonal, Abcam), anti-CD31 (hamster, clone 2H8, Millipore), or anti-NG2 (rabbit polyclonal, Millipore). The sections were washed several times and then incubated for 2 hours at room temperature with the following secondary antibodies: fluorescein isothiocyanate (FITC)-conjugated anti-rat IgG (Jackson ImmunoResearch), FITC-conjugated anti-rabbit IgG (Jackson ImmunoResearch), Cy3-conjugated anti-rabbit IgG (Jackson ImmunoResearch), Cy3-conjugated anti-hamster IgG (Jackson ImmunoResearch), Cy3-conjugated anti-rat IgG (Jackson ImmunoResearch), or Cy5-conjugated anti-rat IgG (Jackson ImmunoResearch). Cell nuclei were counterstained with 4′6-diamidino-2-phenylindole (Invitrogen). Analysis of the samples was performed with a Zeiss LSM 880 microscope (Carl Zeiss).

### Flow cytometry analysis

For flow cytometry analysis, the tumor mass was dissociated into single cells. Prior to antibody staining, red blood cells were removed by the addition of ammonium chloride-potassium lysis buffer (A1049201, Fisher Scientific) for 3 min at room temperature. To distinguish live cells, the cells were processed with Fixable Viability Dye eFluor 450 (65-0863-18, eBioscience) or Fixable Viability Dye eFluor 780 (65-0865-18, eBioscience) on ice for 30 min, followed by treatment with the mouse Fc receptor-binding inhibitor (CD16/31, BD Bioscience, clone 2.4G2) for 15 min at room temperature. The cells were stained using antibodies against CD45 (clone 30-F11, eBioscience), CD3 (clone 17A2, eBioscience), CD3 (clone 145–2 c11, ebioscience), CD8 (clone 53–6.7, eBioscience), CD4 (clone RM4-5, eBioscience), PD-1 (clone eBio4B10, eBioscience), Tim-3 (clone 8B.2C12, eBioscience), Lag-3 (clone C9B7W, eBioscience), CD62L (clone MEL-14, BD Bioscience), CD44 (clone IM7, BD Bioscience), Nos2 (clone CXNFT, eBioscience), F4/80 (clone BM8, eBioscience), Ly6c (clone HK1.4, eBioscience), Arg1 (clone A1exF5, eBioscience), Ly6G (clone 1AB-Ly6G, eBioscience), CD11b (clone M1/70, eBioscience), PD-L1 (clone MIH5, eBioscience), CD11c (clone N418, eBioscience). Flow cytometry was performed using a CytoFLEX flow cytometer (Beckman Coulter), and the resulting statistics were analyzed using Flowjo software (Tree Star, Ashland, Oregon, USA).

### NanoString gene expression analysis

RNA was extracted from tumor tissues using TRIzol (Invitrogen) to perform Nanostring gene expression analysis. RNA quality was verified using a fragment analyser (Advanced Analytical Technologies, Ankeny, Iowa, USA). Immune profiling was performed with a digital multiplexed Nanostring nCounter PanCancer Immune Profiling mouse panel (NanoString Technologies) as previously described. Data analysis was performed using nSolver software (NanoString Technologies). The mRNA profiling data were normalized against the housekeeping gene, and analysis was performed using R software (www.rproject.org).

### RT^2^ Profiler PCR array

After intraperitoneal injection of MC38 colon cancer cells into C57BL/6 mice, the spleens and tumors were harvested 7 and 18 days later, respectively. Mouse spleen and tumor tissues were dissociated into single cells, and CD45^+^CD3^+^ cells were isolated using a MoFlo XDP cell sorter (Beckman). Total RNA was then obtained from CD45^+^CD3^+^ cells using Trizol Reagent (Invitrogen) and cDNA was synthesized using the RT^2^ First Strand Kit (Qiagen, 330404). Finally, T cell anergy and immune tolerance RT^2^ Profiler PCR arrays were performed with synthesized cDNA (Qiagen, PAMM-074Z). The reactions were performed in a LightCycler 96 system (Roche) and the results were analyzed using LightCycler 96 SW V.1.1 software (Roche) according to the manufacturer instructions.

### IDO activity

For protein extraction from tumor tissues and tumor-draining lymph nodes, tissues were homogenized in DMEM supplemented with 10% fetal bovine serum and protease inhibitor cocktail solution (GenDEPOT). The protein was then quantified using the Pierce BCA Protein Assay Kit (Thermo Fisher, 23227)and Kyn expression was measured using the Kynurenine ELISA kit (ImmuSmol, BA E-2200), following the manufacturer’s instructions.

For further information, see online supplemental methods.

10.1136/jitc-2020-002195.supp1 Supplementary data

### Statistical analysis

Statistical analyses were performed using GraphPad Prism V.7.0 software (GraphPad Software, La Jolla, California, USA). The data are presented as the mean±SD. Statistical significance was assessed by unpaired two-tailed Student’s t-tests or or analysis of variance with Tukey post hoc test. Statistical significance was set at p values less than 0.05.

## Results

### Intraperitoneal colon cancer cells established a highly angiogenic and immunosuppressive milieu within the peritoneal cavity

**Figure 1:**
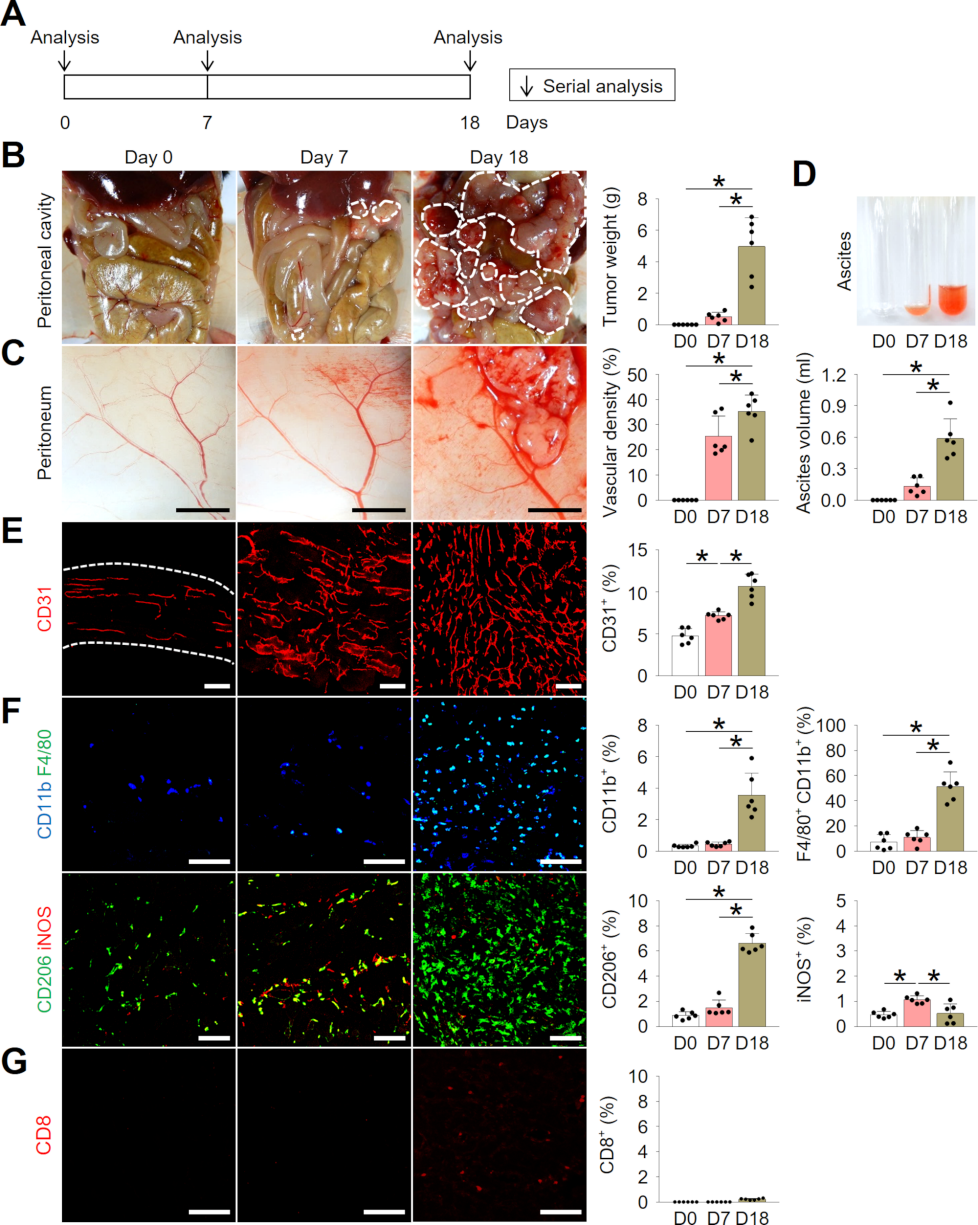
Intraperitoneal colon cancer cells established a highly angiogenic and immunosuppressive milieu within the peritoneal cavity. Mice were intraperitoneally implanted with MC38 colon cancer cells and temporal changes were serially analyzed. (A) Schematic diagram depicting the experimental schedule. (B) Representative images and comparisons of peritoneal tumors. (C) Representative images and comparisons of the peritoneal tumor blood vessels. (D) Representative images and comparisons of malignant ascites over time. (E, F) Representative images and comparisons of CD31^+^ blood vessels (E), and CD11b^+^ F4/80^+^ tumor-associated macrophages (TAMs), CD206^+^ M2-like macrophages, and iNOS^+^ M1-like macrophages (F). (G) Representative images and comparisons of CD8^+^ T cells. Data are pooled from two experiments with n=6 per group (B–G). Values are shown as the mean±SD. *P<0.05; ANOVA with Tukey post hoc test. Scale bar=5 mm (C), 100 µm (E–G). ANOVA, analysis of variance.

To investigate temporal changes in the peritoneal cavity during PCCC, MC38 colon cancer cells were implanted into the peritoneal cavity of C57BL/6 mice (figure 1A). At day 7 after implantation, peritoneal membrane was thickened with dozens of small scattered tumor nodules, accumulating a small amount of ascites from tumor neovessels. At day 18, peritoneal cavity was almost filled with numerable metastatic nodules with a large amount of hemorrhagic ascites (figure 1B–D). Without intervention, median survival of PCCC model was approximately 20 days, therefore, most analyses were performed at day 18.

Histological analysis revealed that PCCC established a highly angiogenic milieu with robust CD31^+^ tumor neovessel formation (figure 1E). Additionally, analyses of immune cells showed that an increasing proportion of F4/80^+^CD11b^+^ macrophages infiltrated the tumor with the progression of PCCC. Among these macrophages, the proportion of CD206^+^ M2-like macrophages was enriched, compared with that of iNOS^+^ M1-like macrophages (figure 1F). Moreover, there were very few CD8^+^ T cell infiltration within peritoneal tumors, indicating PCCC is a non-T cell-inflamed tumor model (figure 1G). Collectively, these findings suggest that colon cancer cells can establish a highly angiogenic and immunosuppressive milieu during the progression of peritoneal metastasis.

### Intraperitoneal colon cancer cells impaired intratumoral lymphocytes by upregulating multiple immune checkpoints

**Figure 2:**
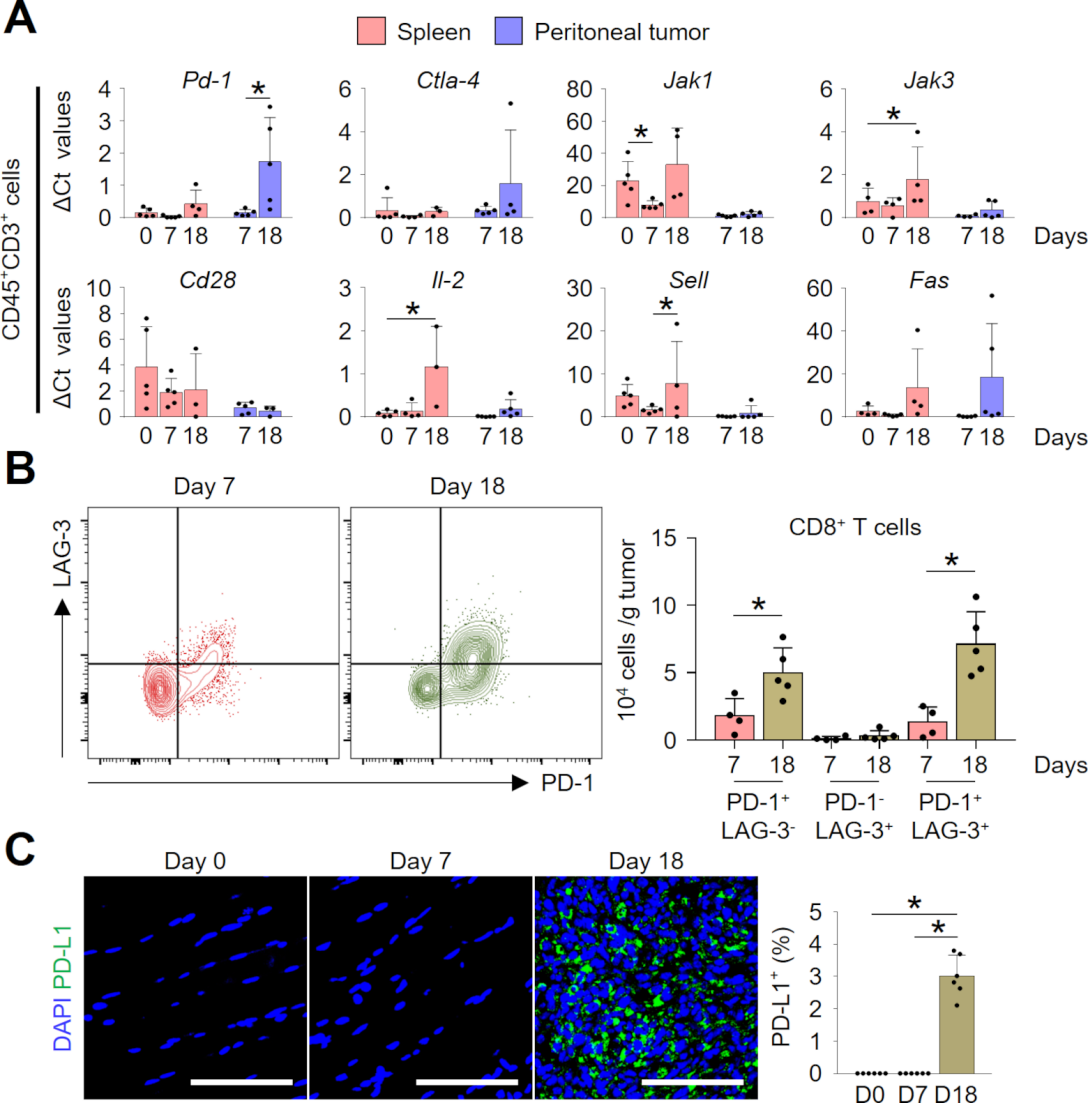
Intraperitoneal colon cancer cells impaired intratumoral lymphocytes by upregulating multiple immune checkpoints. (A) Temporal changes of gene expression levels of splenic and intratumoral CD45^+^CD3^+^ lymphocytes in peritoneal tumor-bearing mice. (B) Representative flow cytometric plot and comparison of intratumoral CD8^+^ T cells expressing PD-1 and/or LAG-3. (C) Representative images and comparisons of tumorous PD-L1^+^ expression over time. Data are pooled from two experiments with n=3–5 per group (A), n=4–5 per group (B), and n=6 per group (C). Values are shown as the mean±SD. *P<0.05; ANOVA with Tukey post hoc test (A, C), Two-tailed Student’s t*-*test (B). Scale bar=100 µm (C). ANOVA, analysis of variance; DAPI, 4′6-diamidino-2-phenylindole.

To examine how the colon cancer cells manipulated lymphocytes within the peritoneal cavity, we isolated T cells and analyzed temporal changes in their phenotypes in the spleen and peritoneal tumor. During the progression of PCCC, intratumoral lymphocytes upregulated immune checkpoint genes, such as *Pd-1* and *Ctla-4*, and downregulated genes involved in T-cell activation and survival (*CD28*, *Il-2, Jak1,* and *Jak3*) (figure 2A). These changes were more apparent in tumor compared with spleen, and in late PCCC (day 18) compared with early PCCC (day 7). Moreover, the expression of *Sell*, a homing receptor for lymphocytes, was evident in the spleen, whereas it was almost undetectable within peritoneal tumors. T cells with terminally exhausted phenotype (PD-1^+^LAG-3^+^CD8^+^ T cells)^29^ were significantly enriched in the TME of the late PCCC compared with that of early PCCC (figure 2B). Finally, intratumoral programmed cell death ligand 1 (PD-L1) expression was also highly upregulated during peritoneal tumor progression (figure 2C). Therefore, our findings indicate that peritoneal colon cancer cells suppress T-cell effector function by inducing various immune checkpoints within peritoneal TME.

### STING activation normalized tumor angiogenesis and immunity during peritoneal dissemination of colon cancer

**Figure 3:**
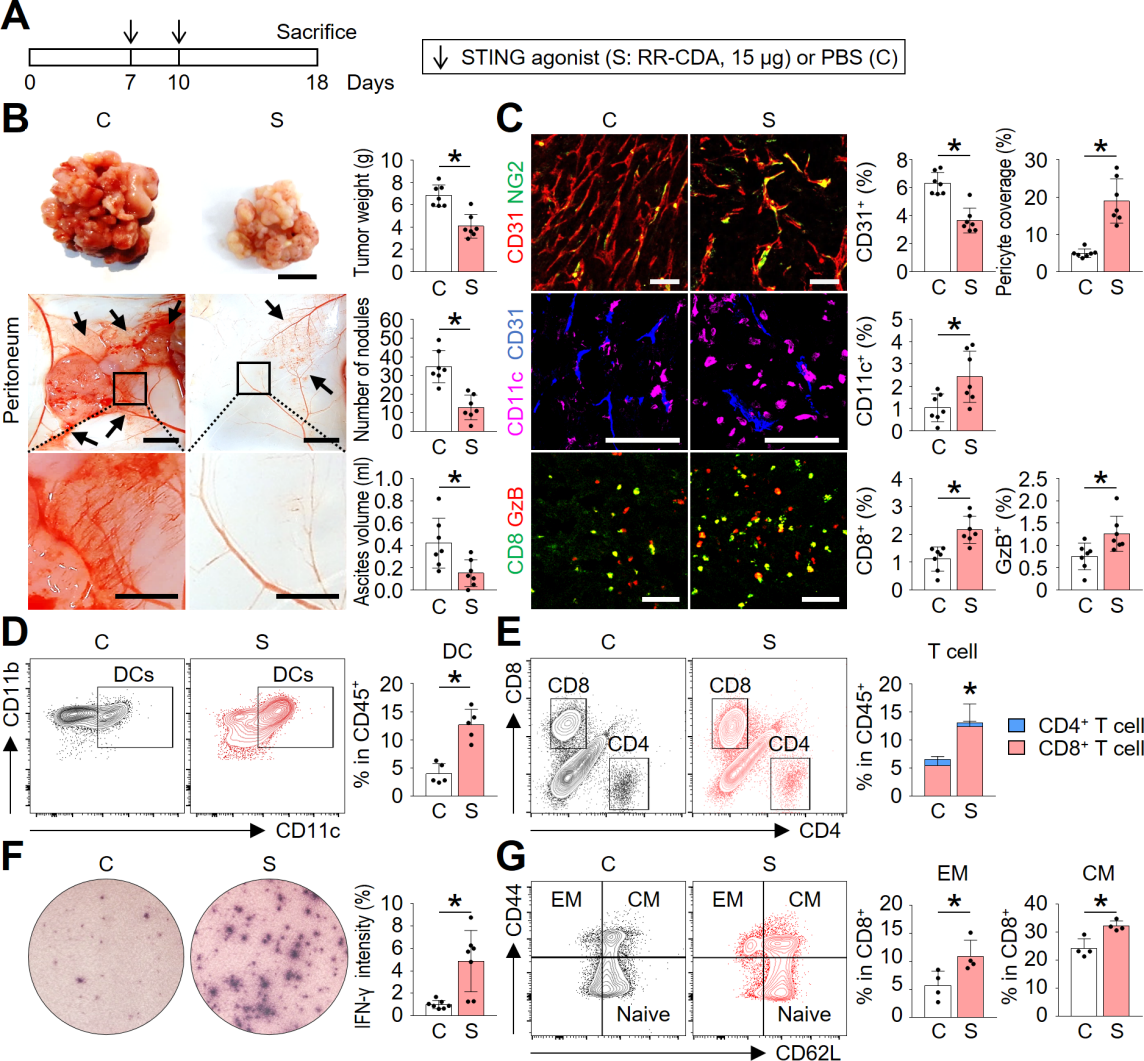
STING activation normalized tumor angiogenesis and immunity during peritoneal dissemination of colon cancer. Mice with PCCC were intraperitoneally treated with phosphate-buffered saline (PBS) or STING agonist. (A) Schematic diagram depicting the treatment schedule. (B) Representative images and comparisons of peritoneal tumor nodules, blood vessels, and malignant ascites in the PBS- or STING agonist-treated mice. (C) Representative images and comparisons of CD31^+^ blood vessels, NG2^+^ pericyte, CD11c^+^ dendritic cells, CD8^+^ T cells, and granzyme B (GzB) within peritoneal tumors. (D) Representative flow cytometric plot and comparison of tumor-infiltrating CD11c^+^ dendritic cells. (E) Representative flow cytometric plot and comparison of tumor-infiltrating CD4^+^ or CD8^+^ T cells. (F) Representative images and comparison of IFN-γ ELISPOT in splenocytes. (G) Representative flow cytometric plot and comparison of effector memory (EM, CD44^+^CD62L^−^) and central memory (CM, CD44^+^CD62L^+^) T cells within the spleen. Data are pooled from two experiments with n=7 per group (B, C, E, F), n=5 per group (D) and n=4 per group (G). Values are shown as the mean±SD. *P<0.05 vs control; Two-tailed Student’s t*-*test. Scale bar=10 mm (B, top), 5 mm (B, middle), 2 mm (B, bottom), and 100 µm (C). IFN-γ, interferon-γ; PCCC, peritoneal carcinomatosis of colon cancer; STING, stimulator of interferon genes.

To overcome the immunologically unfavorable peritoneal microenvironment during PCCC progression, we treated peritoneal tumor-bearing mice with intraperitoneal injections of a potent STING agonist, RR-CDA (also known as MIW815 or ADU-S100) (figure 3A). Through a dose-finding study, we determined 15 µg of RR-CDA as an optimal dose to treat peritoneal tumors (online supplemental figure 1A–C). When injected twice every 3 days, RR-CDA markedly reduced the peritoneal tumor nodules and suppressed the accumulation of malignant ascites in the peritoneal cavity compared with PBS-treated control mice (figure 3B).

To unveil the STING-induced changes in the TME, we histologically analyzed tumor blood vessels and tumor-infiltrating immune cells (figure 3C). First, STING treatment reduced CD31^+^ vascular density by 42.1% and increased NG2^+^ pericyte coverage by 3.8-fold compared with PBS-treated control mice, thereby inducing normalization of the tumor vasculature. Next, STING activation induced a 2.3-fold increase in intratumoral CD11c^+^ dendritic cells, a 1.9-fold increase in intratumoral CD8^+^ T cells, and 1.6-fold increase in granzyme B (GzB)-expressing T cells within the peritoneal tumor, compared with control, suggesting the activation of both innate and adaptive immunity within the peritoneal tumor.

Flow cytometric analyses also revealed consistent findings: 3.2-fold increase in CD11c^+^ dendritic cells and 2.2-fold increase in CD8^+^ T cells compared with the control group, while no changes in CD4^+^ T cells were found (figure 3D, E). Increased CD8^+^ T cells in STING-treated mice revealed stronger IFN-γ activities on stimulation with tumor cells in ELISPOT assay compared with control mice (figure 3F). Finally, lymphocytes from STING-treated mice showed 1.9- and 1.3-fold higher frequency of effect memory (CD44^+^CD62L^-^) and central memory (CD44^+^CD62L^+^) CD8^+^ T cells compared with those form control mice (figure 3G).

To confirm whether the antitumor effects of STING agonist is also valid in peritoneal carcinomatosis of cancers other than colon cancer, we treated female mice with peritoneal carcinomatosis of ovarian cancer (ID8) (online supplemental figure 1D). Two intraperitoneal injections of RR-CDA effectively suppressed peritoneal dissemination of ovarian cancer, and reduced the formation of hemorrhagic ascites. (online supplemental figure 1E, F).

Taken together, these results indicate that STING agonist treatment effectively suppressed the progression of peritoneal carcinomatosis and malignant ascites formation by normalizing tumor vessels and promoting peritoneal antitumor immunity.

### STING activation reprogramed immune phenotype of PCCC

**Figure 4:**
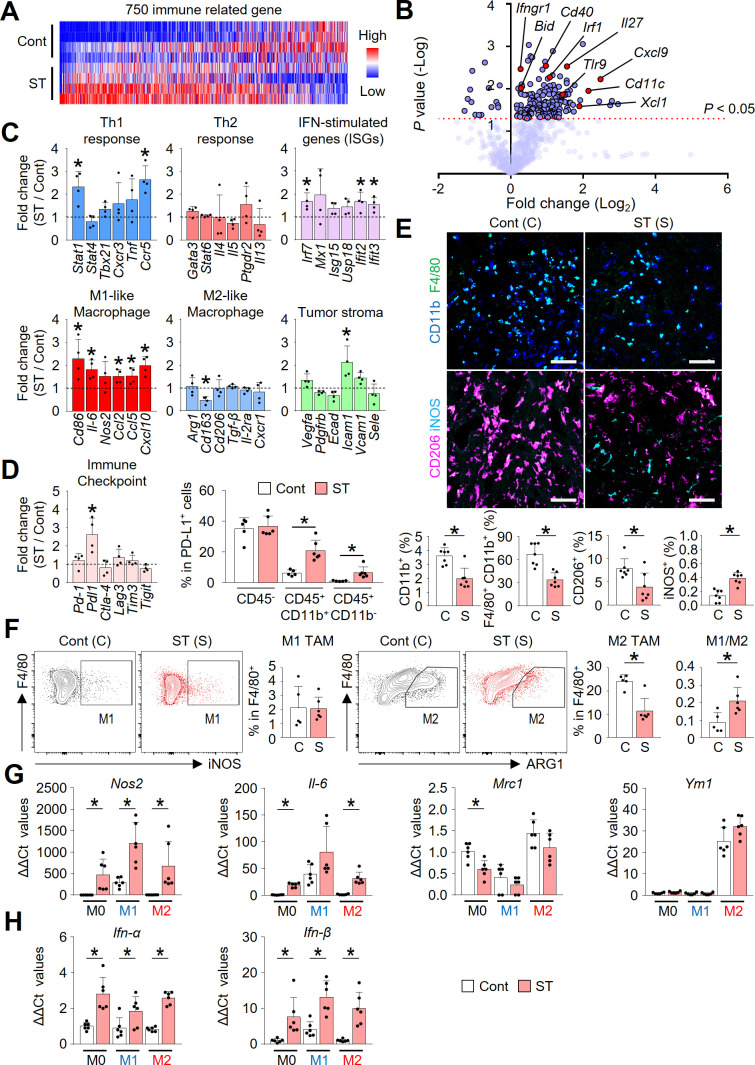
STING activation reprogramed immune phenotype of PCCC. (A) NanoString immune-related gene expression heat map. Red and blue denote upregulated and downregulated genes, respectively. (B) Volcano plot showing the changes of gene expression profiles in STING-activated tumors compared with control tumors. The red line indicates p<0.05. (C) Comparisons of mRNA expression levels for genes related to the Th1 response, Th2 response, IFN-stimulated genes, M1-like, M2-like macrophages, and tumor stroma. (D) Comparisons of transcriptional changes of various immune checkpoints and PD-L1 expression levels in non-immune cells (CD45^-^), myeloid cells (CD45^+^CD11b^+^) and other immune cells (CD45^+^CD11b−) in tumors. (E) Representative images and comparisons of intratumoral CD11b^+^ myeloid cells, CD11b^+^F4/80^+^ TAMs, CD206^+^ M2-like TAMs, and iNOS^+^ M1-like TAMs. (F) Representative flow cytometric plot and comparison of M1-like (F4/80^+^iNOS^+^) and M2-like (F4/80^+^Arginase-1^+^) TAMs in tumors. (G) Comparisons of expression levels of genes related to macrophage phenotypes in bone marrow-derived macrophages (BMDMs) stimulated with PBS or STING agonist. (H) Comparisons of type-I IFNs expression in BMDMs treated with PBS or STING agonist. Data are pooled from two experiments with n=4 per group (A–C), n=4–6 per group (D), n=7 per group (E), n=5–6 per group (F) and n=6 per group (G, H). Values are shown as the mean±SD. *P<0.05; two-tailed Student’s t*-*test. Scale bar=100 µm (E). IFN, interferon; IL2, interleukin 2; PCCC, peritoneal carcinomatosis of colon cancer; PBS, phosphate-buffered saline; STING, stimulator of interferon genes.

Intraperitoneal STING treatment triggered a very rapid type-I IFN response, especially *IFN-β,* which started within 1–4 hours of first RR-CDA administration, followed by dramatic changes in IFN-stimulated genes such as *Isg15, Usp18, Mx1, Mx2, Cxcl10,* and *Ifit3*, within peritoneal TME (online supplemental figure 2A–C). These STING-induced early IFN responses are most evident in intratumoral CD11c^+^ dendritic cells (online supplemental figure 2D). As these initial responses triggered widespread transcriptional reprograming of peritoneal TME, the STING-treated peritoneal tumors became immunologically distinct from control tumors by 10 days after the initial treatment (figure 4A, B). Especially, the expression of genes related to Th1 response, IFN stimulation, and M1-like macrophage phenotype were significantly upregulated in STING-activated tumors (figure 4C). STING activation also induced immune checkpoint *Pdl1*, which was most strongly upregulated in in CD11b^+^ CD45^+^ myeloid cells, while no significant changes were observed in CD45^–^ cells (figure 4D).

Consistent with transcriptional changes, histological analysis also revealed that infiltration of CD11b^+^ myeloid cells and F4/80^+^CD11b^+^ tumor-associated macrophages was significantly suppressed in STING-treated mice compared with control mice (figure 4E). Among intratumoral macrophages, CD206^+^ M2-like macrophages were reduced by 50.3%, whereas iNOS^+^ M1-like macrophages were increased by 2.8-fold, suggesting macrophage repolarization toward the M1 phenotype by the STING treatment. Moreover, flow cytometric analyses also displayed an increasing trend in the M1/M2 ratio (figure 4F). Additionally, STING activation decreased CD11b^+^Ly6G^+^ myeloid-derived suppressor cells in tumors (online supplemental figure S3).

We further analyzed the role of STING signaling in the repolarization of macrophages by stimulating bone marrow-derived macrophages in vitro with RR-CDA (figure 4G). Consistently, STING activation resulted in a significant upregulation of the expression of M1 macrophage markers, *Nos2* and *Il-6*, and downregulation of the M2 marker, *Mrc1* (*Cd206*). These changes were accompanied with the upregulation of *Ifn-α* and *Ifn-β* in all subtypes of macrophages (figure 4H), suggesting the potential role of type-I IFNs during STING-mediated regulation of the peritoneal TME.

### Type-I IFN signaling and CD8^+^ T cells are indispensable during STING-induced peritoneal vascular and immune normalization

**Figure 5:**
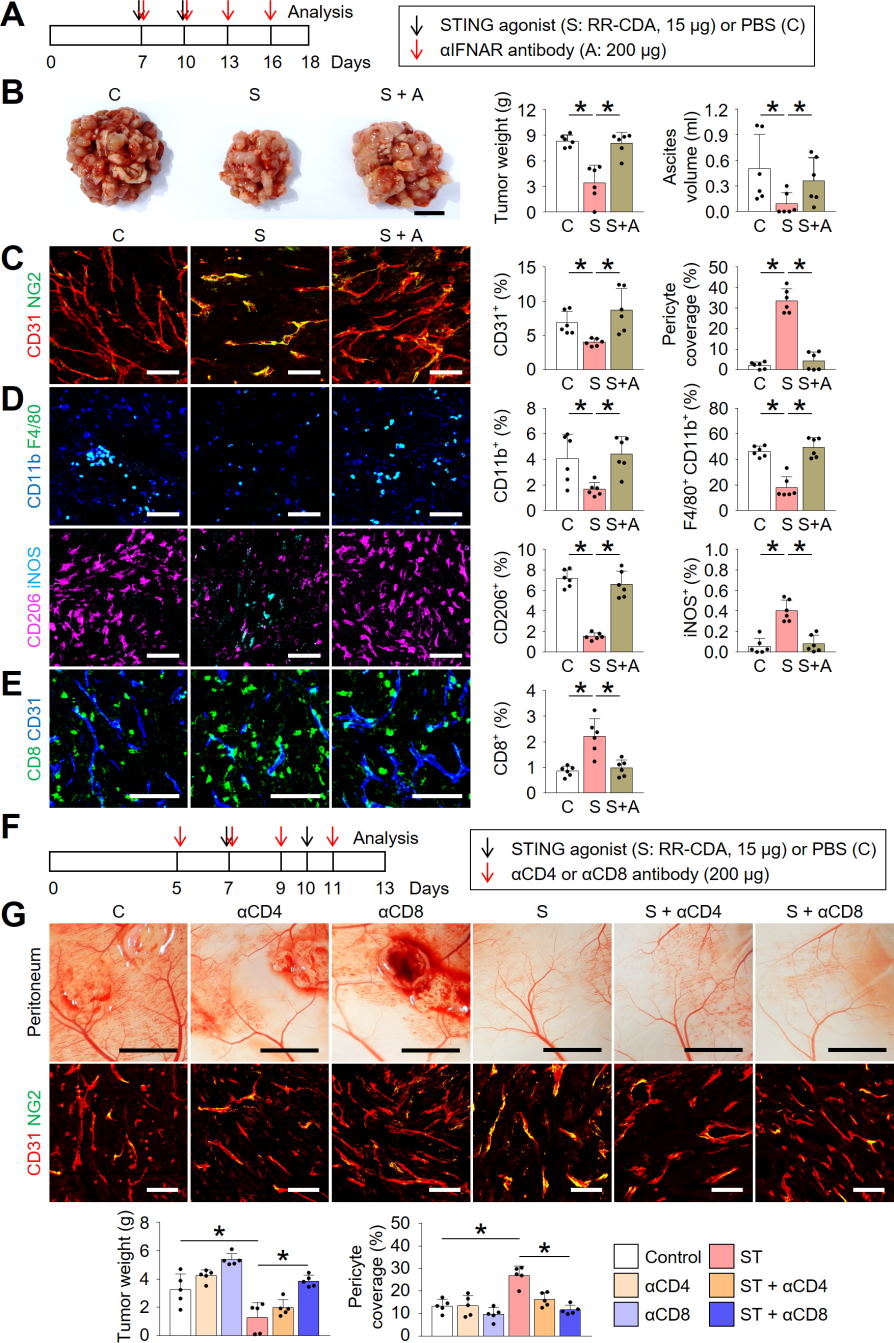
Type-I IFN signaling and CD8^+^ T cells are indispensable during STING-induced peritoneal vascular and immune normalization. Mice were injected with MC38 cells intraperitoneally and treated with STING agonist and/or depleting antibodies against IFNAR (αIFNAR), CD4 (αCD4), or CD8 (αCD8). (A) Schematic diagram depicting the treatment schedule for IFNAR depletion study. (B) Representative images and comparison of the peritoneal tumor burden, and comparison of malignant ascites. (C) Representative images and comparisons of CD31^+^ blood vessels and NG2^+^ pericyte coverages within tumors. (D) Representative images and comparisons of CD11b^+^ myeloid cells, CD11b^+^F4/80^+^ TAMs, CD206^+^ M2-like macrophages, and iNOS^+^ M1-like macrophages within tumors. (E) Representative images and comparison of CD8^+^ T cells within tumors. (F) Schematic diagram depicting the treatment schedule for CD4 or CD8 depletion study. (G) Representative images and comparison of the peritoneal tumor burden, CD31^+^ blood vessels and NG2^+^ pericyte within tumors. Data are pooled from two experiments with n=6 per group (B–E) and n=5 per group (G). Values are shown as the mean±SD p<0.05; ANOVA with Tukey post-hoc test. Scale bar=10 mm (B), 100 µm (C–E), 5 mm (G, top), 100 µm (G, bottom). ANOVA, analysis of variance; IFN, interferon; IFNAR, IFN α receptor; PBS, phosphate-buffered saline; STING, stimulator of interferon genes.

Because STING signaling is a potent inducer of type-I IFN signaling, we questioned whether STING-induced type-I IFN signaling was responsible for the above-mentioned peritoneal vascular and immune remodeling. We treated peritoneal tumors with the STING agonist in the presence or absence of a neutralizing antibody against type-I IFNAR (figure 5A). IFNAR depletion markedly affected the antitumor efficacy of the STING agonist treatment as well as its suppressive effect on malignant ascites formation (figure 5B). Consistently, the blockade of type-I IFN signaling abrogated the antiangiogenic and vascular normalization effects of the STING agonist within peritoneal tumors (figure 5C). Moreover, IFNAR depletion nullified the effect of the STING agonist on macrophages (figure 5D). Finally, interrupting STING signaling by IFNAR depletion also aborted intratumoral infiltration of CD8^+^ T cells (figure 5E).

Since the initial type-I IFN response within TME lasts for several days after STING treatment and it was replaced by long-lasting adaptive immune responses thereafter, we next questioned whether the increased T cells after STING treatment have a role during STING-induced TME remodeling. To determine the roles of T cells, we treated peritoneal tumors with STING agonist in the absence or presence of neutralizing antibodies against CD4 or CD8 (figure 5F). Intriguingly, CD8 depletion substantially negated the antitumor and vascular normalizing effects of STING agonist on peritoneal tumors (figure 5G). Overall, these findings indicate that type-I IFN signaling and CD8^+^ T cells are indispensable for STING-mediated tumor vascular remodeling and antitumor immune response.

### Combination immunotherapy of the STING agonist and anti-PD-1 antibody further enhanced antitumor immunity within the peritoneal cavity

**Figure 6:**
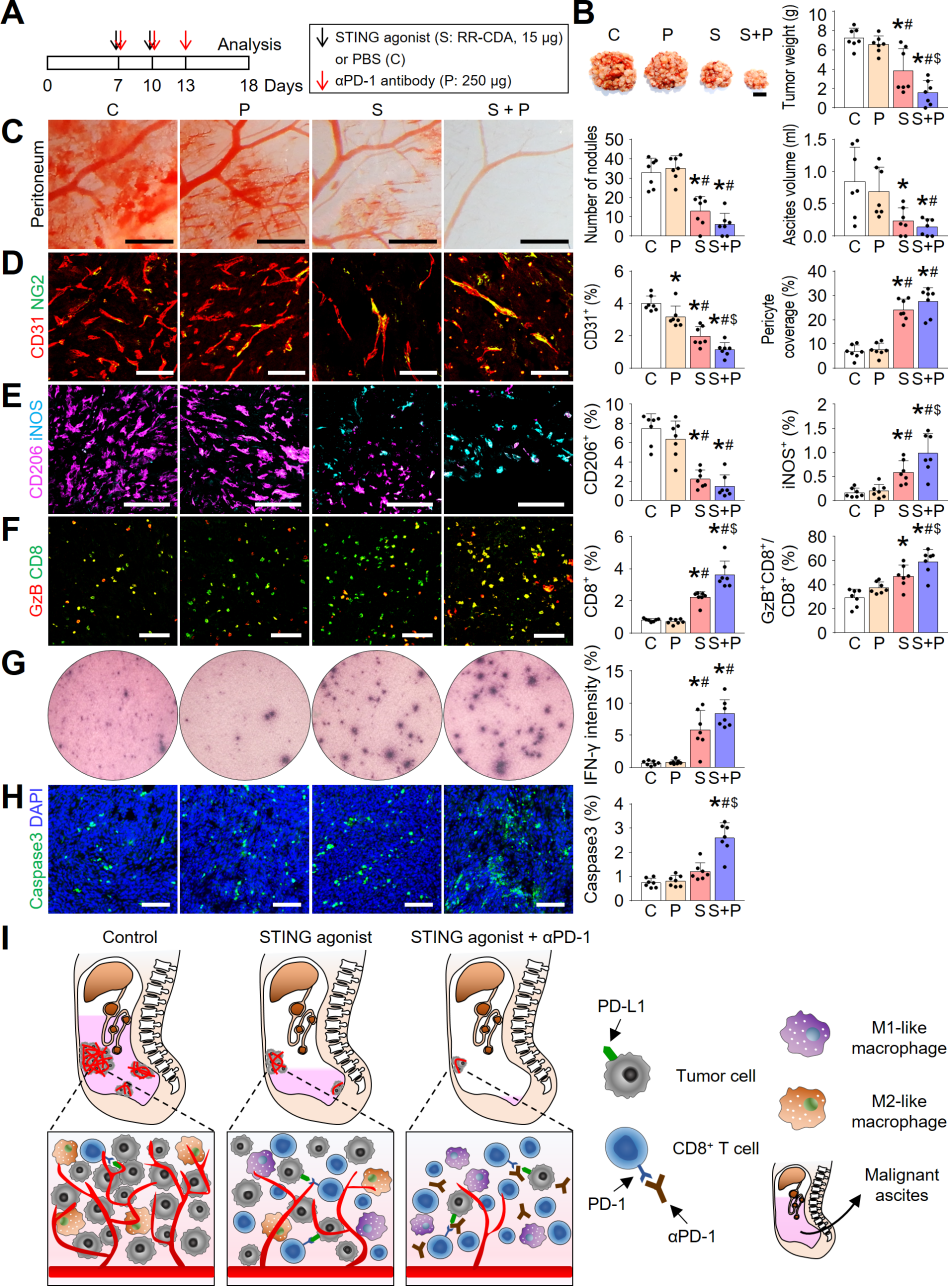
Combination immunotherapy of the STING agonist and anti-PD-1 antibody further enhanced antitumor immunity in PCCC. (A) Schematic diagram depicting the treatment schedule. (B) Representative images and comparisons of the peritoneal tumor burden. (C) Representative images and comparisons of peritoneal tumor nodules and malignant ascites. (D) Representative images and comparisons of CD31^+^ blood vessels and NG2^+^ pericyte coverages within tumors. (E) Representative images and comparisons of CD206^+^ M2-like macrophages and iNOS^+^ M1-like macrophages within tumors. (F) Representative images and comparisons of CD8^+^ T cells and GzB-expressing T cells within tumors. (G) Representative images and comparison of IFN-γ ELISPOT in splenocytes from PBS-treated or STING agonist-treated tumor-bearing mice. (H) Representative images and comparison of caspase 3^+^ apoptotic cells within tumors. (I) Schematic depicting the mechanism by which intraperitoneal STING activation reprograms the peritoneal vascular-immune microenvironment and enhances the antitumor effect of PD-1 immune checkpoint inhibitor. Data are pooled from two experiments with n=7 per group (B–H). Values are shown as the mean±SD. *P<0.05 vs control; #p<0.05 vs P; $p<0.05 vs S; ANOVA with Tukey post hoc test. Scale bar=10 mm (B), 1 mm (C), 100 µm (D–F, H). GzB, granzyme B; IFN-γ, interferon-γ; PBS, phosphate-buffered saline; PCCC, peritoneal carcinomatosis of colon cancer; STING, stimulator of interferon genes.

Although STING monotherapy suppressed peritoneal tumor growth and elicited strong antitumor immunity, the efficacy was not sufficient to fully eradicate peritoneal colon cancer cells. Moreover, we found that repeated treatment with the STING agonist upregulated intratumoral PD-L1 expression (figure 4D). Therefore, we questioned whether simultaneous blockade of the PD-1/PD-L1 axis could further strengthen STING-induced antitumor immune responses. To examine this hypothesis, we treated peritoneal colon tumors with RR-CDA and/or anti-PD-1 antibody (figure 6A). While PCCC tumors were refractory to the anti-PD-1 monotherapy and partially responsive to STING monotherapy, they responded dramatically to the combination treatment (figure 6B). Combination treatment of RR-CDA and anti-PD-1 led to a 78.2% decrease in tumor burden and a 83.4% reduction in the volume of malignant ascites, in some cases resulting in the complete eradication of peritoneal tumors and ascites (figure 6C). Consistently, malformed tumor vasculature was almost completely normalized and the hemorrhagic leakage from these vessels was markedly reduced by the combination treatment (figure 6D). Combination treatment also resulted in a 74.5% decrease in CD31^+^ microvascular density and a 4.0-fold increase in pericyte coverage within peritoneal tumors, suggesting a strengthened vascular normalizing effects mediated by the combination therapy.

Next, we analyzed the immunological effects of combination treatment on peritoneal tumors. Specifically, combination treatment with the STING agonist and PD-1 blockade resulted in a 6.0-fold increase in the proportion of iNOS2^+^ M1-lke macrophages and a 79.9% decrease in the proportion of CD206^+^ M2-like macrophages within peritoneal tumors (figure 6E). Furthermore, combination treatment also resulted in a 4.4-fold increase in the proportion of intratumoral CD8^+^ T cells and a 2.0-fold increase in GzB^+^ activated CD8^+^ T cells within peritoneal tumors, compared with control tumors (figure 6F). Consistently, combination treatment induced a robust IFN-γ secretion from intratumoral T cells (figure 6G). As a result of the enhanced antitumor immunity induced by the combination therapy, intratumoral apoptosis was more evident in tumors treated with combination therapy compared with control tumors (figure 6H).

Given these findings, we found that STING agonist and anti-PD-1 antibody combination could effectively remodel peritoneal TME and suppress peritoneal tumor growth, by inducing vascular normalization and antitumor immunity (figure 6I).

### Concurrent blockade of the STING-responsive COX2 signaling pathway can overcome adaptive resistance to STING treatment and induce long-term survival

**Figure 7:**
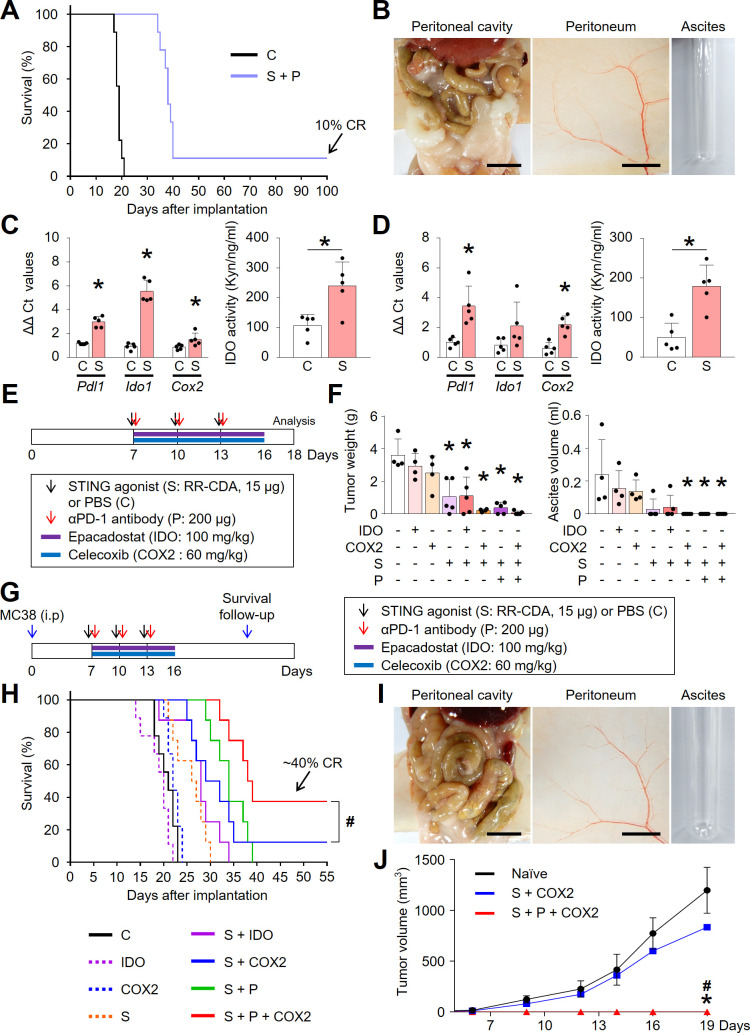
Combination of PD-1 blockade and COX2 inhibitor enhanced STING-induced protective antitumor immunity in PCCC. (A) Kaplan-Meier curves for overall survival. Complete regression (CR) is observed in the combination group. (B) Representative images of abdominal cavity, peritoneum, and ascites in CR mice after dual combination therapy. (C, D) Comparisons of mRNA expression levels of *Pdl1*, *Ido1*, and *COX2* and comparisons of IDO activity in tumor tissues (C) or tumor-draining lymph nodes (D). (E) Schematic diagram depicting the treatment schedule for IDO or COX2 inhibitors. (F) Comparisons of peritoneal tumor burden and malignant ascites. (G) Schematic diagram depicting the treatment schedule for survival analysis and rechallenge. (H) Kaplan-Meier curves for overall survival. ^#^p<0.05, log-rank test. (I) Representative images of abdominal cavity, peritoneum, and ascites in CR mice after triple combination of STING agonist, COX2 inhibitor, and anti-PD-1 antibody. (J) Comparisons of subcutaneous tumor volume in MC38-rechallenged mice. Data are from experiments with n=9 per group (A), n=5 per group (C, D), n=4–5 per group (F), n=8–9 per group (H), n=1–5 per group (J). Values are shown as the mean±SD ^*^P < 0.05 vs control; ^#^p<0.05 vs S+COX2; Two-tailed Student’s *t-*test (C and D) and ANOVA with Tukey post-hoc test (F and J). Scale bar=10 mm (B and I, left), 5 mm (B and I, middle).

Although dual combination therapy with STING agonist and anti-PD-1 antibody prolonged overall survival, most mice experienced recurrence, wherein only a fraction of them (<10%) remained tumor-free and showed durable survival (figure 7A, B), indicating an incomplete survival benefit. Recently, Lemos *et al* reported that STING-induced antitumor immunity can be transient and unstable due to STING responsive pathways, such as IDO and COX2, which act as negative regulators of antitumor immunity.^30 31^ To verify possible IDO and COX2 involvement as resistance mechanisms to STING treatment in the PCCC model, we assessed their expression and activity in peritoneal tumors and tumor-draining lymph nodes (TDLNs) following STING agonist treatment (figure 7C, D). Intriguingly, it was found that STING agonist treatment strongly upregulated both *Ido* and *Cox2* and induced IDO enzymatic activity in both tumors and TDLNs. Therefore, STING activation induced immune regulatory pathways, IDO and COX2, which possibly dampen STING-induced antitumor immunity within the peritoneal cavity.

Therefore, we tested whether concurrent IDO and/or COX2 blockade could overcome these resistance mechanisms and further enhance STING-based immunotherapy (figure 7E, F). While monotherapy with either IDO (epacadostat) or COX2 inhibitor (celecoxib) had no significant effect on tumor growth and malignant ascites, concurrent blockade of COX2 and/or PD-1 further strengthened the antitumor and anti-ascitic effects of STING agonist. However, combining IDO inhibitor to STING agonist did not provide a significant difference in the PCCC model. Consistently, in survival analysis, triple combination of STING agonist with anti-PD-1 antibody and COX2 inhibitor (S+P + COX2) demonstrated superiority over other combinations, showing ~40% complete response (CR) rates (figure 7G, H). Specifically, mice with CR after triple combination therapy remained recurrence-free (figure 7I).

To confirm protective and stable antitumor immunity, we reinjected MC38 tumor cells subcutaneously into the mice which experienced CR of their initial tumors. Mice which experienced CR after triple combination therapy (S+P + COX2) were completely immune to the regrowth of MC38 colon cancer cells and remained tumor-free, whereas mice which experienced CR after dual combination therapy (S+COX2) were not protected from MC38 tumor cell rechallenge (figure 7J). Therefore, mice treated with triple combination immunotherapy acquired protective and stable antitumor immunity.

Collectively, concurrent blockade of regulatory signaling pathways, PD-1 and COX2, enabled STING-induced antitumor immunity to be more potent and durable against peritoneal tumors.

## Discussion

Our present study unveiled microenvironmental changes occurring in the peritoneal cavity during peritoneal dissemination of colon cancer. Colon cancer cells manipulated angiogenesis and immunity to generate an immunologically unfavorable milieu where T cells are scarce and severely exhausted. Intraperitoneal STING activation reversed this situation; it blocked abnormal neovessel formation and restored peritoneal antitumor immunity, thereby establishing a T cell-inflamed microenvironment that could respond favorably to immune checkpoint blockade. Indeed, combination immunotherapy of STING activation and PD-1 immune checkpoint blockade strongly suppressed PCCC and eradicated the accumulation of malignant ascites within the peritoneal cavity.

Malignant ascites is a grave presentation of PCCC, leading to malignant bowel obstruction and malnutrition in patients with colon cancer.^1 2^ Free-floating colon cancer cells in the peritoneal cavity secrete a major pro-angiogenic growth factor, vasular endothelial growth factor A (VEGF-A), and downregulate tight junction-related genes in the peritoneal endothelial layer, thereby increasing the permeability of peritoneal tumor blood vasculature and promoting the accumulation of ascites.^4 10–12 14 32 33^ In this study, we found that intraperitoneal STING treatment effectively reduced aberrant tumor vessel formation and enhanced pericyte coverage of the remaining tumor vessels, thereby suppressing the formation of malignant ascites within the peritoneal cavity. This STING-induced antiascitic effect seemed to be largely dependent on type-I IFN signaling,^23 34–36^ which was previously reported to mutually antagonize VEGF-A/VEGFR2 signaling.^27 35 37^ Moreover, during peritoneal metastasis, STING-activated type-I IFN signaling also suppressed the recruitment of CD206^+^ M2-like macrophages,^34 38–41^ which are known to be involved in the generation of malignant ascites by promoting leaky tumor neovessels within the peritoneal cavity.^24 42 43^ Further studies will be required to gain an understanding on the major sources and targets of type-I IFN on intraperitoneal stimulation of the STING pathway.

Although immune evasion is one of the most important hallmarks of cancer,^44^ its underlying mechanisms during peritoneal metastasis of colorectal cancer have been poorly understood. In this study, we revealed how colon cancer cells disarmed peritoneal immunity during their peritoneal dissemination. First, colon cancer cells promoted aberrant neovessel formation within the peritoneal cavity that induced a massive influx of immunosuppressive myeloid cells, such as CD206^+^ M2-like macrophages. Intraperitoneal colon cancer cells also upregulated the expression of immune checkpoints, PD-L1, PD-1, and CTLA-4, which led to the dysfunction of T cells within peritoneal tumors.^45–47^ Notably, T cells within peritoneal tumors displayed severe immune dysfunction compared with those within the spleen; they upregulated PD-1, as opposed to interleukin-2 and CD28, which are required for T cell activation and survival. Thus, during the peritoneal dissemination of colon cancer, cancer cells manipulated both immune and vascular components of the peritoneal cavity to debilitate peritoneal immunity and generate a protumoral environment.

Intraperitoneal administration of a potent STING agonist that simultaneously targets peritoneal immunity and angiogenesis can overcome this tumor-induced peritoneal immune suppression. STING activation induced type-I IFN signaling,^16 19 20 26 27 48^ which plays a central role in peritoneal antitumor immunity. Type-I IFNs are known to induce potent antitumor effects by activating dendritic cells, promoting cross-priming of tumor antigens to T cells, and enhancing survival of T cells.^16^ In the present study, intraperitoneal STING treatment activated type-I IFN signaling, increased the number of CD8^+^ T cells, and enhanced IFN-γ production from those CD8^+^ T cells.^34 39^ In addition, intraperitoneal STING activation induced the repolarization of tumor-associated macrophages within peritoneal tumors.^36 38 43^ In our study, peritoneal colon cancer cells extensively recruited F4/80^+^CD11b^+^ macrophages, especially CD206^+^ M2-like macrophages, during tumor progression. Remarkably, intraperitoneal STING activation dramatically reduced the number of F4/80^+^CD11b^+^ macrophages and, in particular, decreased protumoral CD206^+^ M2-like macrophage while increasing antitumoral iNOS^+^ macrophages.

Monotherapy with a STING agonist exhibited outstanding antitumor effects by inhibiting peritoneal carcinomatosis and suppressing the accumulation of malignant ascites through T cell influx and macrophage repolarization. However, repeated STING agonist injections strongly upregulated immune checkpoint molecules, such as PD-1 and PD-L1, within peritoneal tumors,^27^ potentially conferring adaptive resistance to the STING immunotherapy. This suggested that simultaneous blockade of these immune checkpoints was needed to maximize and sustain the antitumor efficacy of the STING agonist.^49 50^ Moreover, STING-responsive pathways, such as IDO and COX2, are also known as negative regulators of antitumor immunity.^30 31^ In the present study, we confirmed that these pathways are rapidly upregulated within hours in both peritoneal tumors and lymph nodes following intraperitoneal STING treatment. Furthermore, our study demonstrated that the concurrent PD-1 and COX2 blockade allowed STING agonist to have a superior and more durable antitumor immunity against PCCC as compared with monotherapy. Therefore, future clinical trials are needed to confirm this combination strategy in PCCC patients that do not respond to conventional anticancer therapies.

In conclusion, intraperitoneal STING activation can overcome the highly angiogenic and immunosuppressive peritoneal environment found in PCCC by normalizing both tumor vessels and peritoneal immunity. STING treatment was most effective when combined with PD-1 and COX2 blockade, resulting in the eradication of peritoneal metastasis and malignant ascites.

## Data Availability

Data are available in a public, open access repository. Data are available on reasonable request. All data generated or analyzed during this study are included either in this article or in online supplemental information file. NanoString data were uploaded into GEO database (GSE159825).
