# Supplementary material for: STING activation normalizes the intraperitoneal vascular-immune microenvironment and suppresses peritoneal carcinomatosis of colon cancer
Source: J Immunother Cancer. 2021 Jun 18;9(6):e002195. doi: 10.1136/jitc-2020-002195 (PMC8215239; doi:10.1136/jitc-2020-002195)
Supplement: Supplementary data [file jitc-2020-002195supp001.pdf]

Supplemental information

STING activation normalizes peritoneal vascular-immune milieu and suppresses peritoneal carcinomatosis of colon cancer

Seung Joon Lee, Hannah Yang, Woo Ram Kim, Yu Seong Lee,

Won Suk Lee, So Jung Kong, Hye Jin Lee, Jung Hoon Kim,

Jaekyung Cheon, Beodeul Kang, Hong Jae Chon, Chan Kim

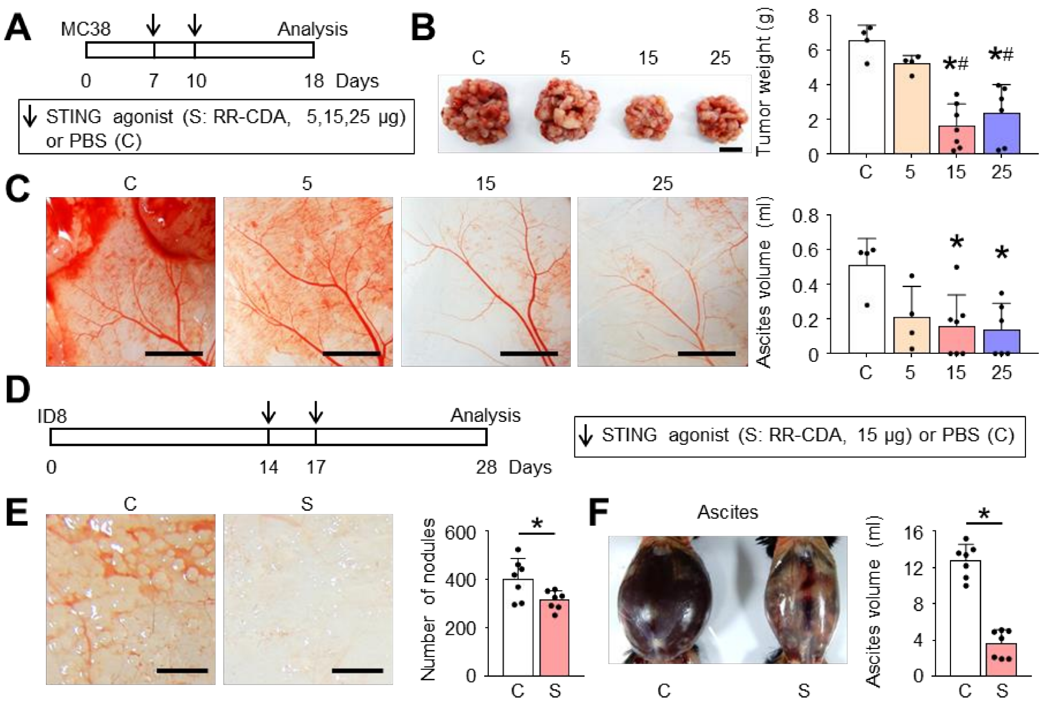

Supplemental figure S1. Intraperitoneal STING treatment suppressed peritoneal dissemination of cancers.

(A) Schematic diagram depicting the treatment schedule for MC38 colon cancers. (B) Representative images and comparisons of peritoneal tumors. (C) Representative images and comparisons of the peritoneal tumor burden and blood vessels. (D) Schematic diagram depicting the treatment schedule for ID8 ovarian cancer. (E) Representative images and comparisons of the peritoneal tumor burden. (F) Representative images and comparisons of ascites in the peritoneal cavity. Data are from experiment with n = 4-7 per group (B, C), n = 7 per group (E and F). Values are shown as the mean ± SD. $^{*}P < 0.05$ versus control; $^{\#}P < 0.05$ versus 5; ANOVA with Tukey post-hoc test (B and C) and two-tailed Student's *t*-test (E and F). Scale bar = 10 mm (B), 5 mm (C), 2 mm (E).

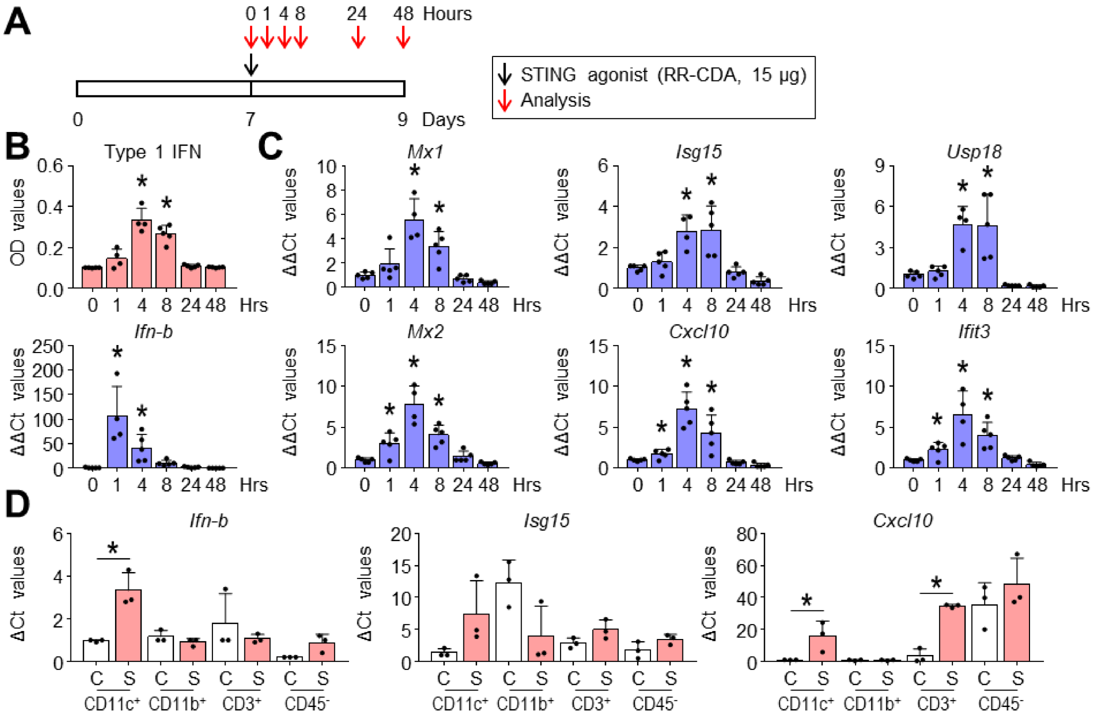

Supplemental figure S2. Early immunologic changes after STING agonist treatment within PCCC.

Mice with PCCC were intraperitoneally treated with phosphate buffered saline (PBS) or STING agonist. Changes in gene expressions were analyzed at various time points.

(A) Schematic diagram depicting the treatment and analysis schedule (B) Temporal changes in *Ifn-b* and type-I IFN activity within tumors at the indicated time points using B16-Blue IFN-α/β reporter cells (InvivoGen). (C) Temporal changes of IFN-stimulated genes (ISGs) within tumors after STING treatment. (D) Gene expressions of *Ifn-b* and ISGs in FACS-sorted dendritic cells ($CD11c^{+}CD11b^{+}CD3^{-}CD45^{+}$), myeloid cells ($CD11b^{+}CD11c^{-}CD3^{-}CD45^{+}$), lymphocytes ($CD3^{+}CD45^{+}$), and non-immune cells ($CD45^{-}$) from peritoneal tumors. Data are from experiment with n = 4-5 per group (B and C) and n = 3 per group (D). Values are shown as the mean ± SD. $^{*}P < 0.05$ versus 0 hours or control; ANOVA with Tukey post-hoc test (B and C) and two-tailed Student's *t*-test (D).

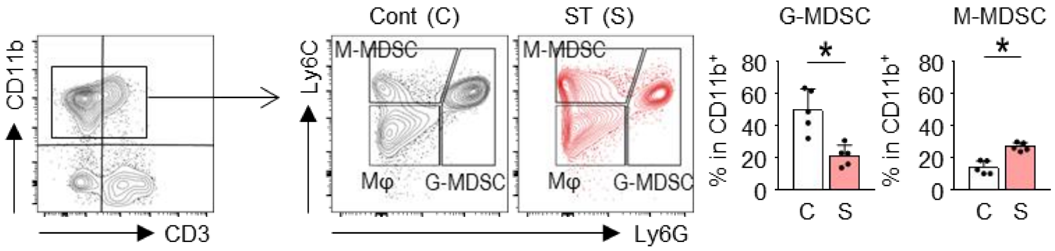

Supplemental figure S3. Representative flow cytometric plot and comparison of myeloid-derived suppressor cells in tumor. Data are from experiment with n = 5 per group. Values are shown as the mean ± SD. *P < 0.05 versus control; and two-tailed Student's t-test.

Supplemental table 1. List of mouse primer sequences used for RT-qPCR

**Target gene**	**Forward sequence 5' – 3'**	**Reverse sequence 5' – 3'**
Nos2	GAG CAC CTT CTT TTC CTT CAT C	TTC ATG ATA ACG TTT CTG GCT CT
Il-6	GGA GGC TTA ATT ACA CAT GTT C	AGT GCA TCA TCG TTG TTC ATA C
Mrc1	CCA CAG CAT TGA GGA GTT TG	CAC AGC TCA TCA TTT GGC TC
Ym1	CAT GAG CAA GAC TTG CGT GAC	GGT CCA AAC TTC CAT CCT CCA
IFN-α	CCT GAG AGA GAA GAA ACA CAG CC	TCT GCT CTG ACC ACC TCC CAG
IFN-β	GCC TTT GCC ATC CAA GAG ATG C	ACA CTG TCT GCT GGT GGA GTT C
Isg15	CAT CC TGG TGA GGA ACG AAA GG	CTC AGC CAG AAC TGG TCT TCG T
Usp18	GGA ACC TGA CTA AGG ACC AGA TC	GAG AGT GTG AGC AGT TTG CTC C
Ifit3	GCT CAG GCT TAC GTT GAC AAG G	CTT TAG GCG TGT CCA TCC TTC C
Cxcl10	ATC ATC CCT GCG AGC CTA TCC T	GAC CTT TTT TGG CTA AAC GCT TTC
Mx1	TGG ACA TTG CTA CCA CAG AGG C	TTG CCT TCA GCA CCT CTG TCC A
Mx2	ACC AGA GTG CAA GTG AGG AGC T	GTA CTA GGG CAG TGA TGT CCT G
Pdl1	TGC GGA CTA CAA GCG AAT CAC G	CTC AGC TTC TGG ATA ACC CTC G
Ido1	GCA GAC TGT GTC CTG GCA AAC T	AGA GAC GAG GAA GAA GCC CTT G
Cox2	GCG ACA TAC TCA AGC AGG AGC A	AGT GGT AAC CCG CTC AGG TGT TG

Supplemental Methods

IFN-γ enzyme-linked immunospot (ELISPOT) assay

To measure the activation of tumor-specific T cells, the spleens were collected from mice administered the STING agonist and/or anti-PD-1 or PBS as a control through intraperitoneal injections. Splenocytes were isolated from the spleen through a 70-μm cell strainer (352350, Corning). The splenocytes were incubated with MC38 cells at a ratio of 1:10 in each well of pre-coated strip plates for 36 hours (37°C with 5% CO_2), and the negative controls were performed by incubating only splenocytes without MC38 cells. The plates were stained with 1 μg/mL of biotinylated anti-mouse IFN-γ antibody (R4-6A2-biotin) for 2 hours, followed by incubation with streptavidin-ALP for 1 hour at room temperature. Finally, after the addition of a substrate solution (BCIP/NBT-plus), the spot density was analyzed using ImageJ software (http://imagej.net/Fiji).

Bone marrow-derived macrophage (BMDM) polarization

Bone marrow cells were extracted from the femurs and tibias of 7–8-week-old C57BL/6 mice. The extracted bone marrow cells were cultured using Iscove’s modified Dulbecco’s medium containing 10% fetal bovine serum, 1% penicillin/streptomycin, and 20 ng/mL granulocyte macrophage-colony stimulating factor (GM-CSF) in 60-mm plates at 37°C with 5% CO_2. After 7 days, BMDMs were harvested and stimulated with 100 ng/mL lipopolysaccharide (LPS; Sigma, L3129) and 20 ng/mL INF-γ (Peprotech, 315-05) to differentiate into M1-like macrophages or with 20 ng/mL IL-4 (Peprotech, 214) to differentiate into M2-like macrophages for 24 hours. To evaluate the effects of STING activation on BMDMs, differentiated BMDMs were treated with 4 μg/mL RR-CDA for 24 hours. The cells were harvested and lysed for further analysis.

Reverse transcription-quantitative polymerase chain reaction (RT-qPCR)

RNA was extracted using TRIzol Reagent (Invitrogen). A Transcriptor first-strand cDNA synthesis kit (Roche, 04 897 030 001) was used to synthesize cDNA, and qPCR was performed using Faststart essential DNA Green Master (Roche, 06 402 712 001). The primer sequences used for qPCR were provided in Supplementary Table 1. LightCycler 96 (Roche) was used for qPCR, and the results were analyzed using LightCycler 96 SW 1.1 software (Roche). Relative fold differences in expression levels were determined using the ΔΔCt method.

Type-I IFN activity

Type-I IFN activity was measured with B16-Blue IFN-α/β cells (InvivoGen) in tumor microenvironment. Tumor tissue were harvested at indicated time points and then were homogenized in DMEM culture medium supplemented with 10% FBS and protease inhibitor cocktail solution (GenDEPOT). B16-Blue IFN-α/β cells were stimulated with 500 μg of extracted protein for 24 hours. Following the manufacturer instructions, type-I IFN activity were measured with QUATI-Blue (InvivoGen).
